# Biological Treatments: New Weapons in the Management of Monogenic Autoinflammatory Disorders

**DOI:** 10.1155/2013/939847

**Published:** 2013-07-21

**Authors:** Antonio Vitale, Donato Rigante, Orso Maria Lucherini, Francesco Caso, Isabella Muscari, Flora Magnotti, Maria Giuseppina Brizi, Susanna Guerrini, Maria Patti, Leonardo Punzi, Mauro Galeazzi, Luca Cantarini

**Affiliations:** ^1^Research Center of Systemic Autoimmune and Autoinflammatory Diseases, Rheumatology Unit, Policlinico Le Scotte, University of Siena, Viale Bracci 1, 53100 Siena, Italy; ^2^Institute of Pediatrics, Università Cattolica Sacro Cuore, Policlinico A. Gemelli, Largo A. Gemelli 8, 00168 Rome, Italy; ^3^Rheumatology Unit, Department of Clinical and Experimental Medicine, University of Padova, Via Giustiniani 2, 35128 Padova, Italy

## Abstract

Treatment of monogenic autoinflammatory disorders, an expanding group of hereditary diseases characterized by apparently unprovoked recurrent episodes of inflammation, without high-titre autoantibodies or antigen-specific T cells, has been revolutionized by the discovery that several of these conditions are caused by mutations in proteins involved in the mechanisms of innate immune response, including components of the inflammasome, cytokine receptors, receptor antagonists, and oversecretion of a network of proinflammatory molecules. Aim of this review is to synthesize the current experience and the most recent evidences about the therapeutic approach with biologic drugs in pediatric and adult patients with monogenic autoinflammatory disorders.

## 1. Introduction

Monogenic autoinflammatory disorders (AIDs) are a recently identified group of hereditary diseases characterized by apparently unprovoked recurrent febrile episodes associated with inflammatory symptoms affecting a host of organs and systems, most often the skin, serous membranes, musculoskeletal apparatus, gastrointestinal tube, eyes, and/or nervous system [1–16]. Differently from the chapter of autoimmune diseases, in monogenic AIDs, the recurrent febrile and inflammatory episodes occur in the absence of autoantibodies or antigen-specific and autoreactive T lymphocytes [17]. Their pathogenesis is mostly related to the presence of mutations in genes encoding proteins involved in the innate immune system regulation or in inflammatory response adjustment, resulting in an immense production of proinflammatory cytokines, in particular interleukin- (IL-) 1 [18].

At a clinical point of view, monogenic AIDs are characterized by considerable heterogeneity in terms of age of onset, frequency and intensity of attacks, clinical manifestations, or responsiveness to treatment, probably due to the wide range of mutations involved in different genes [13–16]. Monogenic AIDs-related mutations can have high penetrance, often generating a more aggressive phenotype, or low penetrance, often underlying a less severe clinical picture with a later onset, lower frequency of attacks, and atypical or paucisymptomatic phenotypes. Therefore, the identification of patients carrying low-penetrance mutations may be problematical, and in these cases there are relevant criticalities in establishing a correct differential diagnosis [19–25]. Our increasing understanding of the molecular mechanisms involved in monogenic AIDs has recently opened new intriguing sceneries in terms of treatment, which should be initiated as early as possible to avoid systemic secondary amyloidosis, which is considered the most dreadful complication of monogenic AIDs, occurring in up to 25% of overlooked patients [26, 27]. 

Aim of this review is to synthesize the current experience and evidences about this ever-new therapeutic approach in monogenic AIDs.

## 2. Classification of the Monogenic Autoinflammatory Disorders

Systemic hereditary monogenic AIDs (see Table 1) include familial Mediterranean fever (FMF), tumor necrosis factor receptor-associated periodic syndrome (TRAPS), the family of cryopyrin-associated periodic syndromes (CAPS), which in turn include familial cold urticaria syndrome (FCAS), Muckle-Wells syndrome (MWS), and neonatal onset multisystem inflammatory disease (NOMID, also named “chronic infantile neurological cutaneous and articular syndrome” or CINCA syndrome), mevalonate kinase deficiency syndrome (MKD), also known in the past as “hyper-gammaglobulinemia D syndrome”, NLRP12-associated autoinflammatory disorder (NLRP12AD), a granulomatous disorder with familial presentation called Blau syndrome (BS), and, finally, hereditary pyogenic disorders, which include Majeed syndrome (MS), PAPA (pyogenic arthritis, pyoderma gangrenosum, and acne) syndrome (PAPAs), and IL-1 receptor antagonist deficiency (DIRA). Some of these—namely FMF, MKD, MS, and DIRA—are transmitted by autosomal recessive inheritance, while the others—TRAPS, FCAS, MWS, NOMID, NLRP12AD, BS, and PAPAs—are autosomal dominant. The genes associated with monogenic AIDs have been identified in recent years and, with the exception of MKD, which is caused by the deficiency of mevalonate kinase, the second enzyme of mevalonate/isoprenoid pathway, encode for proteins involved in the activity of inflammasome, a multiprotein complex which activates the processing and secretion of IL-1*β* and different other cytokines with proinflammatory effects [28]. In addition, MKD is characterized by a 1–8% residual enzymatic activity, while the complete lack of this enzyme causes a distinct metabolic syndrome, called mevalonic aciduria (MA) [29].

## 3. Cornerstones of Treatment in Monogenic Autoinflammatory Disorders 

The main three objectives of therapy for patients with monogenic AIDs are (i) controlling symptoms, (ii) improving patient's quality of life, and (iii) preventing long-term complications. For years, treatment has been symptomatic, based on the sole utilization of nonsteroidal anti-inflammatory drugs (NSAIDs), high-dose corticosteroids, colchicine, or immunomodulators. With the exception of colchicine in FMF, these treatments often fail to provide an adequate control of symptoms and inflammation indexes, in particular serum amyloid-A (SAA), which must be kept within a normal range as the product of its cleavage accumulates progressively in various tissues, giving rise to systemic amyloidosis [30–32]. In general, any treatment should be adapted to maintain SAA concentration within the reference range, and any treatment that fails to guarantee the suppression of SAA levels is to be considered insufficient and must be changed [32]. 

**Figure 1:**
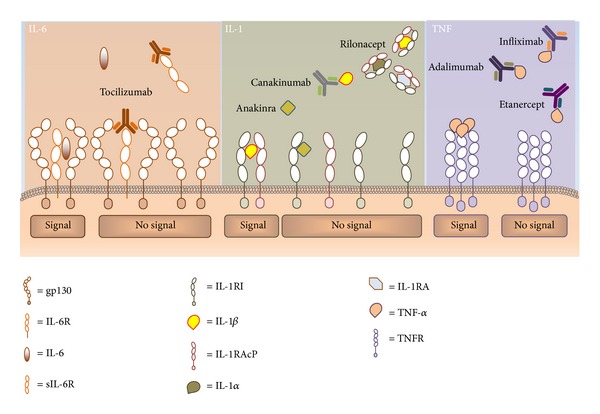
Mechanisms of IL-1, IL-6, and TNF-*α* targeted therapies. Binding of IL-6 to the IL-6 receptor complex, involving IL-6 receptor (IL-6R) and glycoprotein 130 (gp130), leads to activation of IL-6 signal transduction. Tocilizumab, a recombinant humanized anti-IL-6 receptor antibody, inhibits the binding of IL-6 to IL-6R or soluble IL-6R (sIL-6R), thus blocking IL-6 inflammatory response. Binding of IL-1*β* to the IL-1 receptor type I (IL-1RI) promotes a receptor complex formation with the IL-1 receptor accessory protein (IL-1RAcP), that results in signal transduction activation. IL-1-targeted therapy includes anakinra (IL-1R receptor antagonist), canakinumab (anti-IL-1*β* IgG1 mAb), and rilonacept (soluble IL-1 receptor that binds IL-1*β*, IL-1*α*, and IL1RA). Adalimumab, infliximab, and etanercept are anti-TNF blockers. Adalimumab is a fully human monoclonal anti-TNF antibody. Infliximab is a mouse/human chimeric monoclonal anti-TNF antibody. Etanercept is a dimeric fusion protein of TNFR2 (p75) linked with the Fc region of human IgG_1_.

Recently, the introduction of biological pharmaceutical agents has revolutionized the approach to monogenic AIDs, in particular for CAPS and TRAPS, while for FMF, colchicine is still the mainstay of therapy, resulting efficacious in 90–95% of patients [30, 31]. Mechanisms of IL-1, tumor necrosis factor (TNF)-*α*, and interleukin-6 (IL-6) targeted therapies are shown in Figure 1. Obviously, the most appropriate treatment for monogenic AIDs must be tailored to the single patient, based on the severity of his/her clinical phenotype, which can vary greatly [33, 34]. In addition, any treatment efficacy should be evaluated on the basis of routinary control of inflammatory parameters, SAA and proteinuria, to test and treat an eventual subclinical and latent inflammation. The recent and ongoing clinical trials on biologic treatments in the monogenic AIDs are listed in Table 2.

## 4. Familial Mediterranean Fever (FMF)

The mainstay of FMF therapy continues to be colchicine, for its ability to control recurrent attacks as well as to prevent amyloidosis, which is the major long-term ominous complication of FMF [35, 36]. In some cases, proteinuria due to amyloidosis disappears while treatment with colchicine [37]. The initial colchicine dose is 1 mg/day, which may be slowly increased up to 2.5 mg/day in cases of partial response, but in children colchicine dosage can also be calculated based on body weight or body surface area [38]. Recently, a web-based registry in which clinical information on anonymized patients with monogenic AIDs was collected retrospectively as part of the Eurofever initiative (EAHC Project no. 2007332) indicated that 121 patients with FMF received colchicine: 75 (62%) experienced a complete response, 44 (36%) a partial response, and 2 failed to respond [34]. This confirms that true nonresponders to colchicine are very rare and should be distinguished from patients treated with insufficient dosages or those with poor compliance. In fact, compliance is hampered by the frequent occurrence of side effects, particularly at the gastrointestinal level, which sometimes induce patients to abandon colchicine [39, 40]. Corticosteroids seem to be effective in decreasing attack symptoms and pain, while NSAIDs attenuate clinical signs and generally fail to prevent relapses, though they can act as simple analgesics during attacks [41, 42].

Although the role of TNF-*α* in the pathogenesis of FMF is not yet well defined, over the past decade many patients with FMF have been treated with anti-TNF agents. At present, three TNF-*α* antagonists are in use: etanercept, a fusion protein of the TNF receptor and the Fc region of human IgG_1_, infliximab, a chimeric anti-TNF monoclonal antibody, and adalimumab, a fully humanized monoclonal antibody against human TNF-*α*. To date, etanercept is the most used anti-TNF agent in FMF patients, followed by infliximab. In most cases, anti-TNF agents can control FMF attacks quite effectively, improving clinical manifestations and acute phase response [43–51]. In such cases anti-TNF agents led to the improvement of proteinuria in those patients with a secondary amyloidosis: 2 out of 8 patients with FMF-related amyloidosis treated with infliximab have showed complete resolution of proteinuria, or proteinuria improvement in the others as well, suggesting a possible role in the control of renal disease outcome [47–51]. Loss of efficacy for colchicine was described in a 25-year-old woman, which was then treated with infliximab and then with etanercept, who had lost responsiveness to infliximab probably for the production of neutralizing anti-infliximab antibodies [52]. In other cases, anti-TNF agents have brought only a partial benefit, and resolution of symptoms was noted with anti-IL-1 agents [53]. In line with this finding, Ozen et al. reported the effect of anti-TNF therapy (etanercept) and anti-IL-1 treatment (anakinra) in 6 cases resistant to colchicine. Although etanercept lowered the number of attacks (from 3-4 attacks per month to 2 attacks per month), attacks still recurred and acute-phase reactants remained high in 2 patients; thus etanercept was considered ineffective and all patients were switched to anakinra [54]. On the basis of these cases, however, anti-TNF agents seem to be an option for patients with FMF who are unresponsive or intolerant to colchicine therapy and might have a promising role in the treatment of FMF-associated amyloidosis [55].

Recently, in agreement with the more recent studies about the pathogenetic origin of FMF, given that high levels of IL-1*β* (and NF-*κ*B) were considered responsible for most disease manifestations, IL-1 inhibitors, such as anakinra, a competitive IL-1 receptor antagonist, canakinumab, a fully humanized IgG_1_ monoclonal antibody specifically acting against IL-1*β*, and rilonacept, a dimeric glycoprotein consisting of human IL-1 receptor extracellular domains and the Fc portion of human IgG_1_, have been effective in colchicine-resistant patients and are actually regarded as the most valid therapeutic option for FMF patients unresponsive or intolerant to colchicine as well as those with concomitant vasculitis [56–58]. Accordingly, data from the Eurofever registry showed that 3 patients were treated with anakinra, with a complete response in all cases, including 1 patient who failed to respond to colchicine [34]. However, to date, the availability of case series alone and the lack of randomized and controlled studies or cohort studies give to anakinra, along with the remainder of TNF-*α* inhibitors, an evidence level “4” and a “C” recommendation strength.

In March 2013, according to a systematic search of the literature through PubMed/Medline, as many as 29 FMF patients have been treated with anakinra, as described in 16 clinical reports published from 2006 [54, 56, 58–66]. In all cases there was a good clinical response, and anakinra led to the complete disappearance of FMF clinical manifestations and/or normalization of inflammatory markers. Moreover, even in cases of partial response, anakinra led to a significant improvement in terms of frequency of attacks and severity of clinical manifestations. Interestingly, a girl diagnosed both with FMF and Behçet's disease underwent anakinra treatment and had a completely positive clinical response (including Behçet's clinical features): however, although she was symptom-free and her acute-phase reactants remained normal, at the 18th month of treatment, proteinuria gradually increased and serum albumin levels decreased, due to likely amyloidosis which was previously established [61]. In another case, anakinra led to the stabilization of renal function in terms of glomerular filtration rate as well as of proteinuria at the 17-month follow-up visit [60].

In most cases, anakinra dosage was 1-2 mg/kg/day (for pediatric patients) or 100 mg/day (for adult ones), though in 1 case anakinra was administered during FMF attacks, suggesting its possible on-demand employment [62]. Interestingly, in the case presented by Moser et al., the dose of anakinra had to be increased from 100 mg three times weekly to 100 mg daily after renal transplantation, due to the reactivation of FMF, probably related to an improved drug clearance [63]. At last, some clinicians have administered anakinra 100 mg two or three times a week [58, 60, 63] and also every 48 hours [58, 64–66]. The management of colchicine therapy after starting anakinra may vary greatly, with some authors reducing colchicine dosage, others maintaining the same dosage, and others discontinuing colchicine. As regards safety, skin manifestations at the site of injections were the most frequently observed side effect. However, 1 patient experienced acute interstitial pneumonia fifteen days after starting anakinra [64], and another one presented *Rotavirus* gastroenteritis, *Haemophilus* bronchitis, and a slight neutropenia, which did not require hospitalization [60]. Although hypertension is not regarded as an anakinra-related side effect, 1 patient experienced high blood pressure levels requiring antihypertensive therapy [65]. 

To the best of our knowledge, only 4 FMF patients have been treated with canakinumab in the last two years, leading in all cases to a prompt and full resolution of clinical phenotype, corroborating the concepts about efficacy of anti-IL-1 inhibition in FMF patients and suggesting that canakinumab should be considered as a potent alternative option for refractory colchicine-resistant FMF [56, 67, 68]. No side effects were recorded in any of these cases and all of them continued colchicine. Interestingly, 1 patient who had been taking colchicine since the age of 10, but started to have weekly attacks at 14 years, was treated with anakinra but became anakinra-resistant after nine months: this same patient surprisingly responded to canakinumab in only one week, with all laboratory parameters returning to normal [68]. 

With regard to rilonacept, a randomized, double-blind, single-participant alternating treatment study was recently conducted: 14 FMF patients who were unresponsive or intolerant to colchicine received rilonacept or a placebo. Rilonacept significantly reduced the number of FMF attacks and was shown to have an acceptable safety profile, implying its possible role in the treatment of colchicine-resistant FMF [69].

In conclusion, even though none of the anti-IL-1 biological agents are licensed for treatment of FMF and are generally prescribed as off-label drugs, anti-IL-1 agents currently expand the therapeutic choice for the colchicine-resistant or colchicine-intolerant subgroups of patients [55]. In particular, canakinumab can represent an alternative especially in the management of pediatric FMF patients, following its longer half-life and lower frequency of administration, which leads to a better compliance and fewer injection-site side effects. Because of the absence of data regarding long-term efficacy and prevention of amyloidosis, further studies are needed to define safety, tolerance, and side effects of anti-IL-1 agents and analyze whether progression of kidney disease in the case of amyloidosis can be stopped. Meanwhile, colchicine therapy could be continued during anti-IL-1*β* administration to prevent amyloidosis even in colchicine-resistant patients.

## 5. Tumor Necrosis Factor-Associated Periodic Syndrome (TRAPS)

Treatment of TRAPS appears to be more challenging than in other monogenic AIDs due to the considerable genetic heterogeneity and protean clinical spectrum of disease. A few patients gain symptomatic relief from high-dose NSAIDs, while colchicine or immunomodulators such as methotrexate, cyclosporine, and thalidomide produce very little benefit [70]. Inflammatory attacks are often responsive to corticosteroid administration, and patients may need increasing doses if frequent relapses occur, or even long-time administration in order to prevent flares [71, 72]. These subjects may also become prone to metasteroidal comorbidities. Furthermore, corticosteroids do not seem to provide a complete protection from the risk of developing reactive amyloidosis, as they do not normalize SAA levels in the majority of patients [73]. 

Data from the Eurofever project indicated that NSAIDs and corticosteroids were prescribed in 48 and 88 patients, respectively, mainly as on-demand therapy, with favorable results in the majority of cases [34]. In addition, colchicine was beneficial in 21 of 39 patients, 3 of whom had a complete response. The identification of *TNFRSF1A* mutations as the genetic cause of TRAPS raised the possibility that blocking TNF—even though elevated TNF is not observed in most TRAPS patients [74]—could potentially represent the primary therapeutic strategy in TRAPS. Interestingly, patients with the low-penetrance R92Q mutation seemed to respond better to NSAIDs and colchicine versus patients carrying other *TNFRSF1A* mutations [34]. Among biologic agents, to date, the cornerstone of TRAPS treatment has been etanercept, which has been shown to prevent inflammatory attacks and/or to allow the reduction of corticosteroid administration [72, 75–77]. Anecdotal reports describe its efficacy in the treatment of TRAPS-related reactive amyloidosis as well [78]. Bulua et al. have recently shown that although etanercept reduces symptoms and serum inflammatory markers in a dose-dependent manner, it does not completely normalize symptoms or acute-phase reactants in TRAPS patients [79]. In addition, long-term adherence to etanercept may be poor, and a significant number of patients may need to switch to anti-IL-1 therapy, most frequently due to the lack of efficacy and development of injection-site reactions [79]. A progressive decline in responsiveness to etanercept might occur over time in some cases [80, 81], and resistant patients have also been recently reported [82]. These data suggest a nonspecific anti-inflammatory action of etanercept in TRAPS [83]. Accordingly, data from the Eurofever registry showed that etanercept was beneficial in 32 out of 37 patients, even though only 11 (30%) experienced a complete response [34].

In terms of the TNF-*α* neutralizing agents, both infliximab and adalimumab may cause paradoxical inflammatory attacks in TRAPS patients [81, 84]. Their paradoxical effect could be induced by (i) an increase in antiapoptotic activity and oversecretion of proinflammatory cytokines; (ii) more stable binding complexes with soluble TNF and their much higher binding avidity to transmembrane TNF of monoclonal antibodies than etanercept [85]; (iii) a reduced shedding infliximab-bound TNF*α*/TNF receptor from the cell surface, leading to a marked increase in cytokine secretion and increased proinflammatory response [84]. For these reasons, caution is strongly advised when prescribing infliximab and adalimumab in patients with TRAPS.

In etanercept-resistant patients, IL-1 inhibitors have recently been shown to induce a stable and longer lasting effect in controlling TRAPS clinical manifestations and also to obtain a prompt normalization of acute-phase reactants in most patients. Though promising, results obtained with IL-1 antagonists are, to date, limited to few cases and must undergo further evaluation in larger cohorts of patients [25, 80, 86]. Anakinra has recently been shown to prevent disease relapses in the short term and induce a steady disease remission [25, 80]; in addition, its long-term efficacy and safety in patients with and without amyloidosis have also been confirmed [86]. Refractoriness to anakinra has been reported in a patient carrying a T50 M *TNFRSF1A* mutation [87]. We recently reported the first TRAPS patient successfully treated with canakinumab [88]: this patient carried a low-penetrance V95 M mutation, and canakinumab treatment was effective both in bringing about a rapid and complete resolution of clinical manifestations and in normalizing all markers of inflammation within a few weeks. Treatment was well tolerated and at the 6-month follow-up no adverse events were noted. Therefore, we concluded that canakinumab might represent a successful treatment option in the case of refractory TRAPS [88]. On the other hand, long-lasting drugs targeting IL-1 such as canakinumab and rilonacept could preclude the need for daily injections and the relative patient discomfort, which is mainly related to injection-site reactions. A recent phase-II trial conducted on 20 TRAPS patients showed that canakinumab produced a rapid and effective clinical benefit, which was maintained on continued administration: relapses, occurring at a median of 3 months after the last dose, were usually mild or moderate and resolved upon readministration [89].

Finally, since IL-6 levels may be elevated in TRAPS [74], it has been hypothesized that tocilizumab, a humanized monoclonal antibody that binds specifically to both soluble and membrane-bound IL-6 receptors and inhibits IL-6 receptor-mediated signaling, may be an alternative treatment option. A 52-year-old TRAPS patient resistant to etanercept and anakinra recently underwent tocilizumab administration for 6 months: this treatment aborted an evolving acute attack and prevented further inflammatory attacks; in addition, acute-phase reactants promptly decreased to normal values [90]. This case supports the notion of a prominent role for IL-6 in mediating inflammatory attacks in TRAPS, though these preliminary findings need to be confirmed. In order to prevent reactive amyloidosis, treatment of TRAPS must be followed by the persistent normalization of SAA levels. For this reason, close monitoring of SAA levels is recommended to detect their elevation, which may occur even in symptom-free patients as a reflection of the presence of subclinical inflammation [32]. In conclusion, anakinra and etanercept were assessed in cohort studies, achieving a “2b” quality level; their current strength of recommendation grade is “B”.

## 6. Cryopyrin-Associated Periodic Syndromes (CAPS)

Many NSAIDs, immunosuppressant agents and antihistamines, have been proven as generally ineffective in controlling the typical CAPS manifestations; in contrast, high-dose oral corticosteroids and thalidomide have offered some modest improvement, at the price of numerous adverse side effects and metasteroidal comorbidities [91].

Consistent with CAPS' pathogenesis, which is linked to an increased inflammasome activity, uncontrolled caspase-1 activity and subsequent robust production of IL-1*β* [92, 93], anti-IL-1 treatment appears as an ideal therapy both in controlling clinical manifestations and in preventing the development of systemic amyloidosis [94]. To date, three anti-IL-1 agents have been used for treatment of CAPS patients: anakinra, canakinumab, and rilonacept.

Since 2003, daily subcutaneous injections of anakinra have been reported to offer a quick positive effect on clinical and laboratory CAPS manifestations [95–99]. In a prospective study, 18 NOMID patients were selected to receive anakinra 1-2 mg/kg/daily subcutaneously. In all patients, anakinra markedly improved clinical signs and laboratory abnormalities, and magnetic resonance imaging showed improvement in cochlear and leptomeningeal lesions as compared with baseline. Withdrawal of anakinra uniformly resulted in relapse within days, but retreatment led to prompt new improvement. No patient discontinued treatment, and the most common adverse events were injection-site reactions and upper respiratory infections [100]. These results were invigorated by a retrospective review of 22 patients with CAPS, indicating that anakinra had sustained efficacy on dermatologic and articular manifestations and was well tolerated. Anakinra also resulted in resolution of AA amyloidosis-associated nephrotic syndrome in all affected patients [91]. To date there is no approval for anakinra use in CAPS patients, although it continues to be used as an off-label treatment. A bone erosion on the posterior surface of the patella combined with the progression of distal femoral overgrowth and endosteal thinning of both metaepiphyses has been observed in a 13-year-old boy with NOMID treated with anakinra for 6 years [101]. The main limitation of anakinra is its short half-life, which necessitates daily injections, often leading to injection-site reactions and poor patient compliance.

Canakinumab (150 mg in patients weighing more than 40 kg or 2 mg/kg in those weighing 15–40 kg), administered once every eight weeks as a single dose via subcutaneous injection, provides a prompt and sustained clinical efficacy in CAPS patients [102–107]: it has been approved for treatment of CAPS in the USA and Europe since 2009, making it the only biological agent approved in Europe for therapeutic use in CAPS. The first open-label clinical trial for canakinumab was completed in 2008 with 7 CAPS patients and indicated that CAPS is entirely mediated by IL-1*β* and that canakinumab treatment restores a physiological IL-1*β* production [102]. The first double-blind, randomized trial was completed in 2008 and established that treatment with canakinumab 150 mg (or 2 mg/kg for children) every eight weeks was associated with a rapid remission of symptoms in the great majority of patients with CAPS [103]. More recently, 7 pediatric patients (5 children with MWS and 2 adolescents with NOMID) were enrolled in a phase II open-label study: all patients achieved a complete clinical and laboratory response within seven days after the first dose of canakinumab, 2 mg/kg or 150 mg s.c, and responses were reinduced upon retreatment following relapse [104]. More recently, a phase III study conducted by Kuemmerle-Deschner et al. on 166 CAPS patients, including canakinumab-naive and pretreated patients from previous studies, confirmed the foregoing results, as canakinumab provided substantial disease control in children and adults across all CAPS phenotypes [105]. Similar results were obtained in a phase III study on 35 patients conducted by Koné-Paut et al. [106] and in an open-label study on 19 Japanese CAPS patients conducted by Imagawa et al. [107]. Canakinumab might induce an overall complete remission in 75% and a partial remission in 25% of patients, as emerging from the Eurofever study [34]. 

In 2008 rilonacept was the first Food and Drug Administration-approved biologic therapy in the USA for CAPS, specifically for FCAS and MWS, in patients having 12 years of age or older. Initially, improvement in clinical and laboratory features and good tolerability were highlighted by an open-label trial conducted on 5 patients with FCAS [108]. Later, two consecutive phase III studies including FCAS and MWS patients demonstrated that treatment with rilonacept (160 mg weekly via subcutaneous injection) provided a marked and lasting improvement of the clinical picture of CAPS and also normalized SAA levels [109]. Moreover, rilonacept therapy exhibited a generally favorable safety and tolerability profile. In study 1, rilonacept reduced the disease activity score by 84%, as compared with the 13% reduction among patients receiving placebo, and, in study 2, rilonacept was superior to placebo in maintaining the improvement achieved with previous therapy with the same agent. Only injection-site reactions, upper respiratory tract infections, headache, arthralgia, and diarrhea were the most reported side effects on rilonacept. However, 1 patient died after developing sinusitis and meningitis, although study investigators thought that this death was unrelated to rilonacept [109]. Long-term efficacy and safety profile of rilonacept in the treatment of CAPS have recently been evaluated in a 72-week open-label extension study, once again resulting in the improvement of CAPS clinical phenotype and normalization of inflammatory biomarkers. Moreover, rilonacept exhibited a generally favorable safety and tolerability profile in both adult and pediatric patients with CAPS throughout the extended treatment period [110]. 

According to the Eurofever Registry, among the 94 CAPS patients enrolled, 91.5% received at least one anti-IL-1 agent. Anakinra proved to bring about a complete response in 64% of patients and a partial response in 34% [34]. Rare side effects included local skin and anaphylactoid reactions. 

In conclusion, the use of canakinumab and rilonacept in CAPS patients was assessed in randomized and controlled studies, resulting in a quality level of “1b”; their strength of recommendation grade is “A”. Anakinra was assessed in a cohort study, achieving a quality level of “2b”; its strength of recommendation grade is “B”. In this framework, considering that pathogenesis of CAPS is mediated by IL-1 overproduction and in light of the extraordinary clinical effectiveness of anti-IL-1 agents, there is no role for the use of anti-TNF drugs in CAPS patients.

## 7. Mevalonate Kinase Deficiency Syndrome (MKD)

To date, no single therapy has been found to be effective in the totality of MKD patients. Most of these use NSAIDs during febrile attacks, proving only limited benefit [111]. However, according to the Eurofever Registry, the response to NSAIDs was complete in 13% of patients enrolled and partial in 64% [34]. In contrast, many patients benefit from corticosteroid administration, especially when given in high doses at the beginning of an attack [112]. In this case, data from the Eurofever registry showed that corticosteroids induced a complete or partial response, respectively, in 24% and 67% of patients enrolled [34]. Based on the pathophysiology of MKD, statins were thought to be useful, but, in the majority of cases, statins were ineffective in halting the disease course [111, 112]. In addition, a small randomized controlled trial found that simvastatin decreased the number of febrile days in 5 out of 6 patients, but, clinically speaking, these results appeared quite modest [113].

Anti-IL-1 and anti-TNF-*α* agents are reasonable therapeutic alternatives for patients with MKD, as IL-1 and TNF-*α* seem to play a relevant role in acute inflammatory attacks of MKD [114]. With regard to anti-IL-1 agents, the frequency and severity of fever attacks were eliminated or significantly reduced by anakinra treatment in most cases, demonstrating that most symptoms of MKD might be controlled or at least attenuated by anakinra [111, 115–118]. 

Recently, 8 patients with MKD and 3 patients with MA were treated with anti-IL-1-targeting drugs as first-line therapy: 5 patients received anakinra alone, 2 patients received canakinumab alone and, 4 patients started with anakinra and were later switched to canakinumab, in order to obtain a more convenient dosing schedule and to avoid injection-site reactions. A partial remission was obtained in 7 out of 9 patients on anakinra and 3 out of 6 patients on canakinumab; a complete clinical remission was obtained in 1 out of 9 patients on anakinra and 3 out of 6 patients on canakinumab. Moreover, the authors found that the number of febrile days during attacks decreased from five before treatment to three after anakinra and two after canakinumab. A decrease in C-reactive protein and SAA levels was also recorded. The doses of anakinra varied from 1 to 5 mg/kg/day, but 1 patient received anakinra on-demand the first day and for the seven days following an attack, with good results; the doses of canakinumab ranged from 2 to 7 mg/kg every eight weeks in 5 cases, but 1 patient received canakinumab at a dosage of 7 mg/kg every four weeks [119]. In another study by Bodar et al., anakinra induced a partial remission in 1 of 2 patients with MA, but there was no response in the other one. Continuous treatment in 1 MKD patient induced a complete remission for seven months but was stopped due to side effects. Eight patients with MKD received anakinra on-demand, resulting in a substantial clinical response (≥50% reduction in the duration of febrile attacks) with no change in attack frequency [120].

Anti-TNF treatment may also be an effective treatment choice for MKD patients, leading to improvement of both attacks and acute-phase response [121, 122]. However, a complete response to this drug is not always reported [111, 112]. Etanercept is the most used anti-TNF agent, although a small number of patients have been also treated with infliximab and adalimumab with inconsistent results [111, 123]. According to the Eurofever registry, anakinra was effective in 89% of patients, inducing a complete remission in 22%. Etanercept was effective in 65% of patients, with only 1 complete response [34]. In contrast, Shendi et al. reported a case of a 10-year-old girl who experienced prolonged and severe inflammatory attacks when treated with etanercept and later with anakinra [124]. To sum up, biological treatments may be a promising therapeutic approach for patients with MKD, but further studies are required to confirm their beneficial clinical response or establish their long-term efficacy.

## 8. NLRP12-Associated Autoinflammatory Disease (NLRP12AD)

Currently, NLRP12AD treatment is mainly empirical: corticosteroids and antihistamines administered during the winter may lead to a clinical response, thus resulting in the control of clinical manifestations, but also a beneficial effect of NSAIDs has been reported [125, 126]. Recently, 2 patients with NLRP12AD underwent anakinra administration and initially showed a marked clinical improvement; however, a progressive clinical relapse occurred over time, and anakinra treatment was discontinued after 14 months [127]. The authors identified the mechanism responsible for anakinra resistance in the homeostatic cytokine system, since the initial anakinra-induced IL-1 down-modulation was partially counterbalanced by an increase in TNF-*α* serum level, which could in turn circumvent the action of anakinra and eventually lead to the reactivation of IL-1 hypersecretion [127]. Nevertheless, since the crucial role of IL-1 in the pathophysiology of NLRP12AD has been demonstrated, IL-1 blockade continues to be a possible therapeutic choice [127]. In addition, based on the marked increase of serum TNF-*α* and IL-6 levels observed in 2 NLRP12AD patients treated with anakinra, anti-TNF-*α* and anti-IL-6 agents could represent a further therapeutic approach [127]. 

## 9. Blau Syndrome (BS)

No studies are available about the optimal treatment for patients with BS, due to its rarity and heterogeneity in severity and evolution of its expression. The main target is to prevent ocular manifestations, which may be especially severe [128, 129]. On-demand NSAIDs can be effective for pain control, but they have limited efficacy in the prevention of disease progression [130]. Generally, low-dose glucocorticoids can maintain the quiescent stage, but high-dose corticosteroids are necessary in acute flares [131]. Corticosteroid long-term use can become problematical when a maintenance dose of prednisolone ≥10 mg/day is needed for prolonged periods, and patients become prone to metasteroidal comorbidities. In these cases and in corticosteroid-resistant patients treatment with immunosuppressive drugs, such as methotrexate or azathioprine, should be tried. Bupropion was also considered for treatment of BS in the past [132]. If the response to treatment with prednisolone combined with immunosuppressant agents fails to bring about an adequate clinical remission of the disease, a TNF-*α* inhibitor should be added, such as infliximab (5–10 mg/kg every four-six weeks) [133, 134], although an infliximab-resistant patient has been reported [135]. Interestingly, etanercept and adalimumab do not appear to have a similar beneficial effect on the disease [133–138]. In addition, a pediatric patient with BS and etanercept-induced myelopathy has recently been described [136]. 

A further promising therapeutic approach may be related to anti-IL-1 agents. Anakinra in combination with mycophenolate mofetil has been proven to induce both a clear improvement in inflammatory symptoms and normalization of plasma cytokine levels in 1 BS patient [139], although it was ineffective in other cases [135]. Moreover, a recent in vitro study suggests that BS is not a disease primarily mediated by excessive IL-1*β* or other IL-1 direct activity [135]. Nevertheless, more recently a 4-year-old boy diagnosed with BS and suffering from a drug-resistant panuveitis underwent canakinumab administration: soon after the initiation of canakinumab, inflammatory eye signs subsided, and corticosteroid pulse treatment, which had been almost continuous for the previous 6 months, was no longer needed. The same patient showed high expression levels of innate immunity-related genes before starting anti-IL-1 suppression, and almost all of the upregulated transcripts normalized after the first canakinumab injection, suggesting that BS activity might be sustained by abnormal IL-1 production [140]. Finally, a pilot study highlighted the ability of thalidomide to improve clinical symptoms and laboratory findings in 2 BS patients, indicating also a contributing role for NF-*κ*B in BS pathogenesis [141].

## 10. Majeed Syndrome (MS)

Because of the rarity of MS, treatment is empirical. NSAIDs can provide moderate improvement, and corticosteroids are useful in controlling chronic recurrent multifocal osteomyelitis and skin manifestations, but their long-term use in children is limited by metasteroidal comorbidities. In addition, long-term outcome with this strategy has been poor, with marked failure to thrive and permanent joint deformities [10, 142–145]. Recently, Herlin et al. reported a dramatic clinical, laboratory, and radiological improvement with either anakinra or canakinumab in 2 brothers with MS, opening up a new promising therapeutic avenue. Interestingly, in these same 2 patients, the TNF-*α* inhibitor etanercept brought about no improvement, providing new insights about the pathogenesis of MS [146].

## 11. Pyogenic Arthritis Pyoderma Gangrenosum and Acne Syndrome (PAPAs)

PAPAs generally responds only slowly and partially to systemic corticosteroids, which seem to be beneficial for arthritis but less effective in pyoderma gangrenosum [147, 148]. Immunosuppressive therapies, such as cyclosporine, might lead at least to a partial response, while early treatment with sulfasalazine or leflunomide has induced remission in 1 case [11, 147]. As regards anti-TNF*α* agents, the results are inconsistent: infliximab led to a good response in 3 cases but only a poor response in another patient [148, 149]; etanercept induced a complete remission in 2 out of 4 reported patients [148–151]; adalimumab determined a good response in 3 patients [148, 152]. Since PAPAs has been associated with elevated IL-1*β* production [153], targeted therapy with anti-IL-1 agents has been initiated in several PAPAs patients but unfortunately with inconclusive results. Anakinra was completely effective in 3 out of 9 evaluated cases [154–156]. In another case, anakinra initially brought good results but was discontinued due to multiple infections [148]. The same authors described another patient with PAPAs who was only minimally responsive to anakinra [148]. Preliminary data on the response to anakinra administered over several months of 2 other patients was considered encouraging by Shoham et al. [153], but 2 other ones were completely unresponsive to anakinra administration [148, 149]. Recently, a patient carrying a p.Gly258Ala mutation in the *PSTPIP1* gene, diagnosed as having features of a PAPA-like syndrome, was treated with canakinumab, which led to the rapid remission of clinical signs [157]. Finally, to date, all these mentioned biologic agents have a quality level of “4” and a strength of recommendation grade “C” for PAPAs treatment.

## 12. Deficiency of IL-1 Receptor Antagonist (DIRA)

Patients with DIRA respond only partially to high doses of corticosteroids. However, since endogenous IL-1 receptor antagonist is lacking, DIRA patients show a prompt and sustained response to the substitutive treatment with the recombinant IL-1 receptor antagonist anakinra [12, 158–163]. In particular, anakinra was completely effective in 12 out of 14 cases at the dosage of 1–5 mg/kg/day and partially effective in 2 other patients who showed a good clinical response, without reaching a normalized level of acute-phase reactants. Interestingly, these 2 partially-responder patients presented a homozygous deletion of approximately 175 Kb on the chromosome 2q, that included *IL1RN* and five other genes from a cluster of IL-1-related genes, possibly explaining the reduced efficacy of anakinra [12, 164]. However, other patients carrying this deletion have recently been described as completely responsive to anakinra administration [160, 162, 163]. To date, anakinra has a quality level of “1c” and a strength of recommendation grade “A” for treatment of DIRA.

## 13. Conclusive Remarks

**Figure 2:**
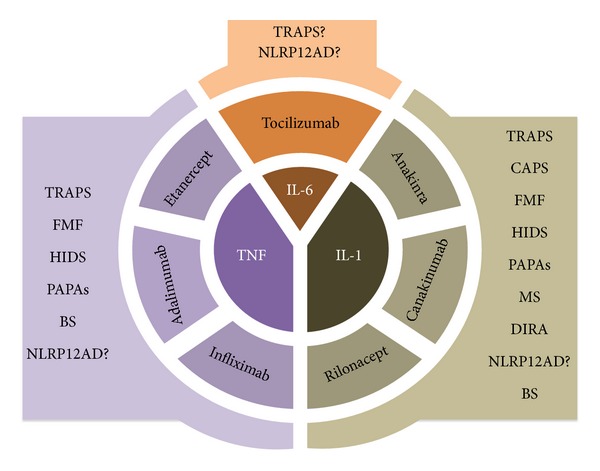
Schematic representation of the biologic agents used in the management of monogenic autoinflammatory disorders. FMF: familial Mediterranean fever; TRAPS: tumor necrosis factor receptor-associated periodic syndrome; CAPS: Cryopyrin-associated periodic syndromes; MKD: mevalonate kinase deficiency syndrome; NLRP12AD: NLRP12-associated autoinflammatory disorder; BS: Blau syndrome; MS: Majeed syndrome; PAPAs: pyogenic arthritis, pyoderma gangrenosum and acne syndrome; DIRA: deficiency of interleukin-1 receptor antagonist.

Due to their hereditary nature, most of monogenic AIDs have an early onset, ranging from the first hours to the first decades of life, but different numbers of patients experience a disease onset during adulthood or go undiagnosed for long periods of time, with recurrent inflammatory symptoms of variable severity remaining misunderstood and bringing a high risk of long-term complications. Even if nowadays there is much more awareness of these disorders, the extreme rarity and relatively recent identification of most monogenic AIDs often result in a delayed diagnosis.  Figure 2 shows a schematic representation of the biologic agents used in the management of the different monogenic AIDs and discussed in this review. The use of biologics requires and dictates that diagnostic times must be anticipated in these disorders, in order to alleviate or suppress many complex clinical phenotypes and avoid the occurrence of secondary amyloidosis. Large scale comparative studies between different monogenic AIDs are needed to establish the best tailored treatment strategy, but probably it will be necessary to translate all discoveries on the immunopathology of these conditions into more effective therapies. In addition, several nonhereditary multifactorial inflammatory diseases presenting clinical similarities with monogenic AIDs and having a hypothetical autoinflammatory pathogenesis might also be managed with a biologic therapeutic approach, opening new perspectives in the battlefield of medicine.

## Figures and Tables

**Table 1:** Basic genetic and clinical keys of the monogenic autoinflammatory disorders discussed in the review.

Disease	*Gene* (Locus)	Protein	Inheritance	Prominent clinical features or potential complications
FMF	*MEFV* (16p13.3)	Pyrin	AR	Recurrent fever, serositis (abdominal and chest pain), arthralgias or arthritides, erysipelas-like skin eruption on the legs, good response to colchicine, and amyloidosis in untreated, resistant, and noncompliant patients
TRAPS	*TNFRSF1A* (12p13)	Tumor necrosis factor receptor 1	AD	Recurrent fever, migrating muscle and joint involvement, abdominal pain, serosal inflammatory involvement, steroid responsiveness of febrile attacks, conjunctivitis, periorbital edema, and amyloidosis
FCAS				Recurrent fever, and cold-induced urticaria-like rash, conjunctivitis, arthralgias
MWS	*NLRP3* (1q44)	Cryopyrin	AD	Recurrent fever, urticaria-like rash, conjunctivitis, arthralgias, sensorineuroal deafness, and amyloidosis
NOMID				Subcontinuous fever, chronic urticaria-like rash, uveitis, papilledema, deforming arthritides involving large joints (knees), aseptic chronic meningopathy, sensorineuroal deafness, and amyloidosis
MKD	*MVK* (12q24)	Mevalonate kinase	AR	Recurrent fever, polymorphous rash, arthralgias, abdominal pain, diarrhea, lymph node enlargement, headache, splenomegaly, oral and genital aphthosis, and high rate of self-resolution during adulthood
NLRP12AD	*NLRP12* (19q13)	Monarch-1	AD	Recurrent fever after cold exposure, arthralgias, and cold-induced urticaria-like rash
BS	*NOD2* (*CARD15*) (16q12.1–13)	NOD2	AD	Intermittent fever, granulomatous dermatitis with ichthyosis-like changes, granulomatous polyarthritis, recurrent panuveitis, and onset before 5 years
MS	*LPIN2* (18p11.31)	Lipin 2	AR	Recurrent multifocal osteomyelitis, dyserythropoietic anemia, and chronic dermatosis
PAPAs	*PSTPIP1* (15q24–q25.1)	CD_2_ antigen-binding protein 1	AD	Pyoderma gangrenosum, cystic acne, and sterile pyogenic oligoarthritis
DIRA	*IL1RN * (2q14)	Interleukin-1 receptor antagonist	AR	Neonatal onset-multifocal osteomyelitis, periostitis, neonatal onset-pustular rash, and dramatic response to anakinra

FMF: familial Mediterranean fever; TRAPS: tumor necrosis factor receptor-associated periodic syndrome; FCAS: familial cold autoinflammatory syndrome; MWS: Muckle-Wells syndrome; NOMID: neonatal onset multisystem inflammatory disease; MKD: mevalonate kinase deficiency syndrome; NLRP12AD: NLRP12-associated autoinflammatory disorder; BS: Blau syndrome; MS: Majeed syndrome; PAPAs: pyogenic arthritis, pyoderma gangrenosum, acne syndrome; DIRA: deficiency of interleukin-1 receptor antagonist; AR: autosomal recessive; AD: autosomal dominant.

**Table 2:** Recent and ongoing clinical trials on biologic treatments in the monogenic autoinflammatory disorders.

	Phase	Status	Study	Disease	ClinicalTrials.gov identifier
Anakinra	III	Not yet recruiting	Kineret (Anakinra) in adult patients with colchicine-resistant familial Mediterranean fever	FMF	NCT01705756
	I	Completed	The use of kineret (anakinra) in the treatment of familial cold urticaria	FCAS	NCT00214851
	II	Recruiting	Anakinra to treat patients with neonatal onset multisystem inflammatory disease	NOMID	NCT00069329
	II	Recruiting	Anakinra for inflammatory pustular skin diseases	PAPAs	NCT01794117

	III	Terminated	Canakinumab to treat neonatal-onset multisystem inflammatory disease	NOMID	NCT00770601
	III	Recruiting	Efficacy, safety, and tolerability of ACZ885 in pediatric patients with the following cryopyrin-associated periodic syndromes: familial cold autoinflammatory syndrome, Muckle-Wells syndrome, or neonatal onset multisystem inflammatory disease	CAPS	NCT01576367
	III	Completed	The safety and efficacy of canakinumab in patients aged 4 years or older diagnosed with cryopyrin-associated periodic syndromes (CAPS) in Canada	CAPS	NCT01105507
	III	Completed	Efficacy and safety study of canakinumab administered for 6 months (24 weeks) in japanese patients with cryopyrin-associated periodic syndromes followed by an extension phase	CAPS	NCT00991146
	II	Active, not recruiting	Evaluation of the safety and efficacy of canakinumab in pediatric patients with colchicine intolerant or colchicine-resistant familial Mediterranean fever (FMF) (CONTROL FMF)	FMF	NCT01148797
	II	Completed	Efficacy and safety, of canakinumab in patients with colchicine-resistant familial Mediterranean fever	FMF	NCT01088880
Canakinumab	III	Recruiting	Efficacy, safety and tolerability of ACZ885 in pediatric patients with the following cryopyrin-associated periodic syndromes: familial cold autoinflammatory syndrome, Muckle-Wells syndrome, or neonatal onset multisystem inflammatory disease	CAPS	NCT01302860
	III	Completed Has results	Efficacy and safety of ACZ885 in patients with the following cryopyrin-associated periodic syndromes: familial cold autoinflammatory syndrome, Muckle-Wells syndrome, or neonatal onset multisystem inflammatory disease	CAPS	NCT00685373
	III	Terminated	Canakinumab to treat neonatal-onset multisystem inflammatory disease	NOMID	NCT00770601
	II	Recruiting	Canakinumab for pyoderma gangrenosum		NCT01302795
	—	Recruiting	Clinical outcomes and safety: a registry study of ilaris (canakinumab) patients (B-confident)	CAPS	NCT01213641
	II	Recruiting	Canakinumab in patients with active hyper-IgD syndrome	MKD	NCT01303380
	II	Completed	Safety, efficacy, pharmacokinetics, and pharmacodynamics of ACZ885 in patients with NALP3 mutations and clinical symptoms	CAPS	NCT00487708
	III	Completed Has results	Efficacy, safety, and tolerability of ACZ885 in patients with Muckle-Wells syndrome (REMITTER)	MWS	NCT00465985
	II	Active, not recruiting	Efficacy and safety study of ACZ885 in patients with active recurrent or chronic (Tumor Necrosis Factor) TNF-receptor-associated periodic syndrome (TRAPS)	TRAPS	NCT01242813
Rilonacept	II	Completed Has results	Rilonacept for treatment of familial Mediterranean fever (FMF)	FMF	NCT00582907
	III	Completed Has results	Rilonacept for treatment of cryopyrin-associated periodic syndromes (CAPS)	CAPS	NCT00288704
	II	Completed	Safety and tolerability of rilonacept in Muckle-Wells syndrome (MWs) or Schnitzler syndrome (ACCILTRA1)	MWS	NCT01045772
	II	Recruiting	Rilonacept for deficiency of the interleukin-1 receptor antagonist (DIRA)	DIRA	NCT01801449
	II	Completed	Interleukin-1 trap to treat autoinflammatory disease	FMF, CAPS	NCT00094900

FMF: familial Mediterranean fever; TRAPS: tumor necrosis factor receptor-associated periodic syndrome; FCAS: familial cold autoinflammatory syndrome; MWS: Muckle-Wells syndrome; NOMID: neonatal onset multisystem inflammatory disease; MKD: mevalonate kinase deficiency syndrome; PAPAs: pyogenic arthritis, pyoderma gangrenosum, acne syndrome; DIRA: deficiency of interleukin-1 receptor antagonist.
